# Analysis of Plantar Fasciitis Videos on YouTube: Quality and Reliability Assessment

**DOI:** 10.7759/cureus.78993

**Published:** 2025-02-14

**Authors:** Ahmet Burak Satılmış, Tolgahan Cengiz

**Affiliations:** 1 Orthopaedics and Traumatology, Taşköprü State Hospital, Kastamonu, TUR

**Keywords:** discern score, health information, jama score, plantar fasciitis, youtube

## Abstract

Objective

Plantar fasciitis is one of the most common causes of heel pain and affects a significant portion of the population. Digital platforms such as YouTube play an essential role in patients' searches for health information. However, the accuracy and reliability of the information shared on these platforms are often questioned.

Method

In this study, the first 50 videos searched for "Plantar Fasciitis" on YouTube were evaluated using DISCERN and JAMA scoring systems. Videos were categorized according to uploaders (physicians, physiotherapists, independent users, etc.) and content types (general information, exercise, non-surgical treatment). Video Power Index (VPI) and statistical analyses were applied to evaluate the quality of the content.

Results

74% of the videos were uploaded by non-physicians, and the DISCERN and JAMA scores of the content uploaded by physicians were statistically higher (p<0.01). However, in the overall evaluation, most of the videos were found to be of low quality. The average length of the videos was 7.63 minutes, and most of the content was shared by physiotherapists (46%).

Conclusion

Most YouTube videos about plantar fasciitis contain low-quality content. Although videos uploaded by physicians appear more reliable, a general lack of information can lead to misinforming patients. Healthcare professionals, universities, and institutions should be encouraged to produce accurate educational content. Improving the information quality of digital platforms will help patients make informed decisions.

## Introduction

One of the most frequent causes of heel discomfort is plantar fasciitis, which arises from degenerative irritation of the plantar fascia origin at the medial calcaneal tuberosity and associated perifascial tissues [1]. It is 7% prevalent in the community and accounts for about 80% of cases [2, 3]. Plantar fasciitis is characterized by sharp, excruciating pain that frequently flares up at the most inopportune moments, such as right before an activity begins or first thing in the morning. Although this discomfort usually goes away with time, it can occasionally become persistent and interfere with a patient's day-to-day activities. It is still unclear what causes plantar fasciitis, one of the sneaky causes of heel discomfort. Nonetheless, it is a condition linked to several risk factors, such as advanced age, elevated body mass index (BMI), excessive use, Achilles strain, calcaneal sprain, pes planus, pes cavus, and a lack of plantar flexor flexibility (reduced ankle dorsiflexion) [4].

Plantar fasciitis can be treated using a wide variety of methods. Orthotic devices, stretching exercises, physical therapy, anti-inflammatory drugs, and foot pads are examples of conservative treatment approaches. Surprisingly, up to 90% of patients respond well to these conservative therapies [5]. In recalcitrant cases, fascia can be released surgically; success rates for this procedure vary from 70% to 90% [6]. However, there is a chance of complications, such as problems with soft tissue healing, superficial infections, or even arch collapse, because of the pressure placed on the plantar fascia area. Over the years, minimally invasive treatments have been developed to address this illness and lower the likelihood of these serious complications. These alternatives include radiofrequency nerve ablation (RFNA) directed at the plantar fascia, extracorporeal shockwave therapy (ESWT), and injections (e.g., steroids or platelet-rich plasma) [6-9].

Patients' quality of life would decline, and their pain would unavoidably become chronic if they did not receive the proper care. Patients, therefore, frequently look for information to have enough knowledge about the illness and to find out about suitable and various treatment alternatives. Internet usage is growing in society and has become a vital source of health information [10, 11]. One of the biggest video-sharing websites in the world is YouTube. To learn about their conditions and the best ways to treat them online, patients and/or their family members frequently view YouTube videos [12]. However, since no control system exists and anyone can upload any video, YouTube may contain inaccurate or deceptive information [11, 13]. As a result, the accuracy and dependability of medical information found online are questioned. This study assessed Plantar Fasciitis using the DISCERN and JAMA rating system to determine the dependability of videos posted on the YouTube sharing platform.

## Materials and methods

Ethics committee permission was unnecessary because the study was conducted as an open-access video study on YouTube. On December 12, 2024, two specialists entered "Plantar Fasciitis" into the YouTube search bar (YouTube LLC, San Bruno, CA, USA). We evaluated the 50 most viewed videos, including the term "plantar fasciitis" in their titles. Exclusion criteria included videos in languages ​​other than English, repetitive content, advertisements, and videos shorter than 30 seconds. The research categorized videos based on criteria such as image type, uploaders, and content categories, which included general information, nonsurgical treatment, exercise training, and massage. Physicians, chiropractors, physical therapists, fitness instructors, and hospital channels were among the categories of uploaders. Essential data were noted for each video, including views, upload date, comments, likes, dislikes, and length. The formula used to calculate Video Power Index (VPI) values ​​(representing the popularity of videos) is like ratio × view ratio/100 [14].

The DISCERN tool, created by the British Library and University of Oxford staff, helps evaluate the caliber of YouTube videos about health. Each of the 15 questions in the DISCERN instrument is scored on a 5-point scale, adding up to a total score ranging from 15 to 75 points, representing the caliber of the data. There are two sections to the DISCERN questions. The first section of the DISCERN instrument (questions 1-8) evaluates the publication's credibility. Questions 9 through 15 in the second section of the DISCERN instrument examine the applicability of various treatment strategies. For DISCERN scores, the following classification scheme is used: scores between 63 and 75 are classified as "excellent," scores between 51 and 62 as "good," scores between 39 and 50 as "average," scores between 28 and 38 as "poor," and scores below 28 as "very poor." Higher scores indicate information quality (Table 1) [15]. Authorship, Attribution, Disclosure, and Currency are the four criteria that make up the JAMA rating system. Each receives one point for four points (Table 2) [16].

**Table 1:** DISCERN Scoring System

Section	Questions	No	Partly			Yes
Reliability of the publication	1. Explicit aims	1	2	3	4	5
	2. Aims achieved	1	2	3	4	5
	3. Relevance to patients	1	2	3	4	5
	4. Source of information	1	2	3	4	5
	5. Currency(data) of information	1	2	3	4	5
	6. Bias and balance	1	2	3	4	5
	7. Additional sources of information	1	2	3	4	5
	8. Reference to areas of uncertainty	1	2	3	4	5
Quality of information on treatment choices	9. How treatment works	1	2	3	4	5
	10. Benefits of treatment	1	2	3	4	5
	11. Risk of treatment	1	2	3	4	5
	12. No treatment options	1	2	3	4	5
	13. Quality of life	1	2	3	4	5
	14. Other treatment options	1	2	3	4	5
	15. Shared decision making	1	2	3	4	5

**Table 2:** JAMA Scoring System

		Rating	
Section		Yes	No
Authorship	Authors and contributors, their affiliations, and relevant credentials should be provided	1	0
Attribution	References and sources for all content should be listed clearly, and all relevant copyright information should be noted	1	0
Disclosure	Website “ownership” should be prominently and fully disclosed, as should any sponsorship, advertising, underwriting, commercial funding arrangements or support, or potential conflicts of interest	1	0
Currency	Dates when content was posted and updated should be indicated	1	0

The average number of views per day was calculated by dividing the total number of views that the observers observed throughout the video evaluation by the number of days that passed between the viewing date and the YouTube video's upload date. Each film was seen concurrently by the same group of observers, who separately noted the JAMA (Journal of the American Medical Association) and DISCERN (Quality Criteria for Consumer Health Information) scores. After that, an average score was determined. The average DISCERN score is calculated as (DISCERN score of the first observer + DISCERN score of the second observer) / 2. The average JAMA score is the sum of the first and second observers' scores divided by two.

Statistical analysis

The study data underwent analysis using the SPSS 26.0 statistical package program (IBM Corp., Armonk, NY, USA), and the results were expressed in numbers, percentages, mean ± standard deviation, median, minimum, and maximum values. Based on the normality test results, the Mann-Whitney non-parametric test was used to compare the mean DISCERN and JAMA scores between the physicians and non-physicians. The association between DISCERN and JAMA scores was evaluated using Spearman correlation analysis, which classified correlation coefficients as weak (r: 0-0.24), moderate (r: 0.25-0.49), strong (r: 0.50-0.74), and very strong (r: 0.75-1.0). Interobserver agreement was evaluated with Cronbach's α, where values ​​<0.5 were deemed unacceptable, 0.5 ≤ α <0.6 as poor, 0.6 ≤ α <0.7 as acceptable, and 0.7 ≤ α <0.9 as excellent. Statistically significant differences were determined at p < 0.05.

## Results

Two of the 50 videos were animated, and 48 featured real images. Physiotherapists shared 23 videos (46%), compared to 13 (26%) from doctors. Examining the video content, 12 movies included fitness training, and 23 videos supplied general information (Table 3). The shortest posted video was 1 minute and 2 seconds, while the longest was 21 minutes and 27 seconds. The most watched video was 13 minutes and 57 seconds long, and the number of views was 9,737,361. According to the sharing date, the most recent post was made 11 months ago, while the oldest was made in 2010. Table 4 shows the distribution of video features by uploaders, including video lengths, views, comments, likes, and dislikes.

**Table 3:** General features of the videos

Image type	N (%)
Real	48 (96%)
Animation	2 (4%)
Uploaders	
Physician	13 (26%)
Health Channel	2 (4%)
Physical Therapist	23 (46%)
Hospital Channel	2 (4%)
Fitness Coach	4 (8%)
Chiropractor	6 (12%)
Video Content	
General Information	23 (46%)
Non-surgical Treatment	6 (12%)
Exercise Training	12 (24%)
Surgery	5 (10%)
Examination	4 (8%)

**Table 4:** Parameters of videos

	Number of Videos	Video Length (minutes)	Like	Dislike	Comment
Physician	13 (26%)	7.05±7.31	23768±20438	783±746	875±1196
Health channel	2 (4%)	6.47±7.03	8977±6938	340±9.19	203±197
Physical therapist	23 (46%)	9.28±8.57	23050±18560	506±314	999±705
Hospital channel	2 (4%)	6.3±4.45	6143±1139	410±128	316±103
Fitness coach	4 (8%)	7.57±3.46	71771±119123	1756±2674	2441±4063
Chiropractor	6 (12%)	3.41±4.51	13930±8107	484±209	771±442

DISCERN, JAMA, and Video Power Index (VPI) scores assigned by observers were analyzed to evaluate potential differences between physicians and non-physicians. The mean VPI, JAMA, and DISCERN values ​​of the videos shared by physicians were not statistically significantly different by two independent viewers (P>0.05). The average of the two observers who saw the videos was included in the statistical analysis to get the DISCERN, JAMA, and Video Power Index (VPI) values. The results showed that the Video Power Index (VPI) in doctor-uploaded movies was 96%, the JAMA score was 2.75, and the DISCERN score was 46. In films posted by non-physicians, the Video Power Index (VPI) was 95%, the DISCERN score was 37, and the JAMA score was 2.35. This analysis demonstrates that the content of films posted by physicians was determined to be of higher quality (p<0.01) when compared to non-physician uploaders. A strong and statistically significant correlation (r: 0.911, p <0.001) was found when DISCERN scores were examined using Spearman correlation analysis, suggesting that the two observers had excellent agreement (Cronbach α = 0.978). Likewise, a substantial and statistically significant correlation (r: 0.463, p < 0.001) was found by Spearman correlation analysis for JAMA scores, suggesting that the two observers had excellent agreement (Cronbach α = 0.968). High rater agreement and consistency were observed in both the DISCERN and JAMA scores.

## Discussion

Approximately 70% to 90% of heel pain cases can be treated conservatively. Nonetheless, 10% to 30% of individuals might need surgery or more invasive procedures [17]. Elevated vascularity, elevated ground substance protein, localized fibroblast overgrowth, and damaged collagen fibers are some of the histological characteristics of this disorder. Furthermore, nonspecific indicators of inflammation in plantar fasciitis have been observed in several studies. Examined etiologically, nerve lesions are one of the many causes of chronic plantar heel pain. As a result, many therapies can be applied to other disease systems. In the current technology era, patients are compelled to look online due to the vast array of offered treatments. The effectiveness and dependability of videos on YouTube, the most popular video platform on the internet, were examined in our study.

As of October 2023, 5.3 billion people worldwide, or 65.7% of the world's population, were active Internet users, and 4.95 billion used social media [18]. Many patients, primarily young males, search for orthopedic information online. Burrus et al. found that 64.7% of patients who used the Internet expressly sought information about orthopedics [19]. YouTube is the most widely used website for informational videos. Nonetheless, there has been discussion in the literature regarding the reliability and correctness of internet-based health information [20]. YouTube orthopedic videos frequently lack an editorial process, which raises questions regarding the accuracy of the data. There is still a lack of trustworthy and accurate information about medical issues available to internet users. Orthopedic surgeons need to be able to comprehend and assess YouTube resources because they give them the knowledge they need to help patients and educate them while navigating the difficulties of illness treatment. 96% (n=48) of the 50 movies examined for our study had genuine footage, whereas 4% (n=2) of the videos were animated. Physicians submitted all animated videos. Most of the studied films were posted by non-physician individuals (n=37, 74%). Physical therapists contributed 46% of the content, the most common contributors among the other non-physician participants, followed by chiropractors (12%), fitness trainers (8%), health channels (4%), and hospital channels (4%). Doctors uploaded 13 (26%) videos. 72% of the 50 films in a different study about rotator cuff tears were posted by non-physicians, underscoring the critical role that non-medical sources play in spreading this specific orthopedic problem. Furthermore, 16% of the videos were animated, and these animations are frequently found on health channels [14]. In keeping with earlier research, which found that average video durations ranged from 6.59 to 7.56 minutes [14, 21], this study's average video length was 7.63 minutes, consistent with the literature.

As we have observed, patients increasingly use social media to research their medical concerns. With over 2 billion subscribers, YouTube remains the most popular online video platform. Numerous problems in the upper extremities, shoulder, knee, hip, and spine have had their video quality evaluated [22-24]. For instance, the video content of one of the foot-related conditions, hallux valgus, was assessed [25, 26]. Like our study, Yüce et al. evaluated the caliber of YouTube videos about calcaneal spurs and plantar fasciitis. Our findings and those of this investigation confirmed the lack of quality, information substance, and reliability [27].

In our study, doctors supplied 26% of the videos. Considering the inter-observer average DISCERN score, the doctor-uploaded movies had the lowest "average" quality. However, neither the website nor the individual user's uploaded films were of high or superior quality. The fact that the JAMA score of the videos that the doctors in our study submitted was noticeably higher than that of the footage that the website uploaded further supports this scenario. Furthermore, our research's statistical analysis reveals that the substance of the movies published by doctors had higher quality (p<0.01) when compared to those submitted by non-physicians. Similarly, Sajadi and Goldman found that healthcare organizations or experts shared 64% of the valuable videos [28]. Şahin et al. discovered that independent user-uploaded movies were of worse quality than those produced by medical professionals [29].

Our findings highlight how crucial the source is when looking for films on YouTube that provide health-related information. When searching, one should take into account the video's source, and doctors should let their patients know how important the video source is. Encouraging and supporting academics, doctors, universities, and professional associations to create videos that offer objective, helpful, instructive, and accurate medical information is crucial. This study has certain drawbacks. YouTube videos were analyzed in a single snapshot, but because of their dynamic structure, new videos are added, viewed, and commented on over time so that the search results could vary. Other study limitations include assessing only English-language videos and using a single keyword. Geographical location is recognized to have an impact on YouTube search results. As a result, YouTube offers a variety of video lists to internet users.

## Conclusions

The study's findings showed that most YouTube videos about plantar fasciitis were of poor quality and did not offer enough trustworthy information. Videos provided by doctors and other healthcare experts were shown to have superior information quality. Such excellent information, however, made up a very modest percentage overall. Most films posted by independent sources and non-physician users raise the possibility of deceiving patients by giving inaccurate or insufficient information. For patients to obtain correct information and medical professionals to be more involved in this field, the video source must be considered. Academic institutions, medical professionals, and universities should close this information gap by creating correct instructional materials and assisting patients in making educated decisions. Otherwise, poor-quality content could cause delays in treatment and misunderstandings regarding health. This situation once again reveals the responsibility of digital platforms in health information.
